# Differential impact of plant-based selenium nanoparticles on physio-biochemical properties, antioxidant defense system and protein regulation in fruits of huanglongbing-infected ‘Kinnow’ mandarin plants

**DOI:** 10.3389/fpls.2024.1476497

**Published:** 2024-11-07

**Authors:** Muhammad Ikram, Naveed Iqbal Raja, Azza H. Mohamed, Zia-Ur-Rehman Mashwani, Ahmad A. Omar, Hassan Gharibi, Roman A. Zubarev

**Affiliations:** ^1^ Department of Botany, Pir Mehr Ali Shah Arid Agriculture University, Rawalpindi, Punjab, Pakistan; ^2^ Department of Biomedical, Surgical and Dental Sciences, Milan State University, Milan, Italy; ^3^ Department of Agricultural Chemistry, College of Agriculture, Mansoura University, Mansoura, Egypt; ^4^ Biochemistry Department, Faculty of Agriculture, Zagazig University, Zagazig, Egypt; ^5^ Citrus Research and Education Center, University of Florida, IFAS, Lake Alfred, FL, United States; ^6^ Department of Medical Biochemistry and Biophysics, Karolinska Institutet, Stockholm, Sweden; ^7^ Department of Pharmaceutical and Toxicological Chemistry, Medical Institute, RUDN University, Moscow, Russia

**Keywords:** *Citrus reticulata* L. Kinnow, mandarin, nanobiotechnology, huanglongbing, selenium nanoparticles

## Abstract

Huanglongbing disease (HLB) is the most severe citrus disease destroying *Citrus reticulata* L. ‘Kinnow’, the most commonly grown mandarin in Pakistan. It is caused by *Candidatus* Liberibacter bacterial species and it spreads through the sucking Asian citrus psyllid insect. The current study was designed to investigate the potential impact of plant extract mediated selenium nanoparticles (SeNPs) on antioxidant defense system, fruit quality and protein regulation in the fruits of HLB-infected ‘Kinnow’ mandarin plants. Garlic cloves extract was used as reducing and capping agent for the synthesis of SeNPs. Various concentrations of SeNPs (25, 50, 75, and 100 mg L^−1^) were exogeneously applied to HLB-positive citrus plants. SeNPs at the concentration of 75 mg L^-1^ affected positively fruit physio-biochemical parameters, e.g., peel thickness, peel weight, fruit weight, fruit diameter, total soluble solids, juice volume, ascorbic acid content and reduced total acidity. Furthermore, SeNPs also enhanced the amounts of total protein and total sugar as well as elevated antioxidant enzymes, e.g., superoxide dismutase, peroxidases, and catalases. Non-enzymatic antioxidant content, e.g., total phenolic and total flavonoids, was also elevated. Proteomics analysis revealed that exposure to SeNPs at the concentration of 75 mg·L^–1^ significantly altered in HLB infected mandarin fruting plants the expression of proteins associated with transcription, protection, cell wall biogenesis, cell wall organization, reproduction, stamen formation, embryo development, inflorescence development, as well as translation and response to oxidative stress. Our results revealed that foliar application of SeNPs influences the protein contents positively, therefore ameliorating fruit physio-biochemical quality by boosting antioxidant defense systems of HLB-infected ‘Kinnow’ mandarin plants.

## 1 Introduction

Biotic stress causes huge damage to plants as well as their derived products and raises the risk of hunger worldwide (Moustafa-Farag et al., 2019). Plants are continuously affected by the biotic stresses in the agricultural and natural environments, which threatens their antioxidant defense system, growth and productivity (Shelake et al., 2024). Plants respond to biotic stresses through the activation of molecular pathways, including pathogen-associated molecular pattern which triggers plant immunity and activates stress-resistance proteins (Medina-Puche, 2024). Huanglongbing (HLB) is regarded as one of the worst biotic diseases of citrus plants, making it a significant problem for the citrus industry worldwide (Pandeya and Shresthab, 2023). HLB disease is caused by the gram-negative bacteria genus *Candidatus* Liberibacter (*C*L). Three species are known to cause the HLB symptoms: *C*L americanus (*C*Lam), *C*L africanus (*C*Laf) and *C*L asiaticus (*C*Las). Among these the American and Asians species can be transmitted through *Diaphorina citri* Kuwayama, commonly known as the Asian citrus psyllid (Limayem et al., 2024). Citrus industry in Pakistan is drastically impacted by HLB disease, with the disease incidence rate of 53% in Bhalwal and the highest disease severity index (50%) in Shahpur (Iqbal et al., 2024). Moreover, it is estimated that in Florida the HLB disease affects 74% of citrus trees, causes annually a more than $1 billion damage and a loss of roughly 5000 jobs (Li et al., 2020; Singerman and Rogers, 2020).

HLB infected trees exhibit canopy reduction, twig dieback, discoloration of leaves, chlorotic patches and mottling, making infected parts look different from the healthy plants (Hussain et al., 2019). Moreover, HLB symptomatic fruits are smaller, occasionally asymmetrical, containing small, misshapen, aborted brownish or black seeds (Xu et al., 2022). Symptomatic mandarin fruits exhibit lower concentrations of sugars, malic acids, soluble solids/acids ratio and higher amounts of titratable acids (TA). Furthermore, in the infected fruits the flavor volatiles valencene, decanal, and ethyl butanoate are lower, and the levels of various monoterpenes are higher. The diseased fruits contain more secondary metabolites in the pulp and orange peel, including limonin, nomilin, narirutin, hesperidin and hydroxycinnamic acids, and their fruit juices lack in sweetness (Dala-Paula et al., 2019).

There is no cost-effective biocompatible treatment available to control the HLB infection in ‘Kinnow’ mandarin plants. Many chemicals-based pesticides that are commercially available are not safe for crops because of the toxicity to humans, biodiversity loss, and decreased soil fertility (Pitino et al., 2020). The application of nanomaterials is a novel strategy in agriculture that lowers the usage of agrochemicals, suppresses disease infestation and enhances plant yield under environmental stresses (Ganachari et al., 2019; Khan et al., 2017; Khan et al., 2023). Selenium (Se) is an essential micronutrient involved in several physiological processes in plants (Gui et al., 2022). Selenium affects plant responses to biotic stress by influencing the sulfate absorption pathway and forming Se-containing organic molecules (Chauhan et al., 2019). Selenium at low quantities ameliorates nitrogen metabolism and photosynthesis processes by suppressing effects of various biotic and abiotic factors (Ghorai et al., 2022). Selenium nanoparticles (SeNPs), due to their small size (≈10–100 nm) and high solubility, are considered pivotal antimicrobial and antioxidant agents to overcome the risks associated with the use of bulk Se (Zohra et al., 2021).

Garlic (*Allium sativum* L.) belonging to the family *liliaceae* has been commonly known as characteristic aroma vegetable with health benefits. Many studies revealed a significant reduction of the risk of chronic diseases, e.g., cancer and cardiovascular disorder, associated with the use of garlic (Sahidur et al., 2023). Garlic also possesses antimicrobial and antioxidant potential due to the presence of polyphenols and organosulfur compounds, phenolic acids and anthocyanins (Putnik et al., 2019). Garlic is also known as a source of selenium. In the current study, garlic cloves extract was used as a reducing, capping and stabilizing agent for the biological synthesis of SeNPs. Garlic clove extracts contain phenolic and flavonoid compounds that can act as reducing and capping agents in the synthesis of garlic based SeNPs (Anu et al., 2017)

SeNPs are potent agents, as they can penetrate the cellular and sub-cellular membranes and interact with seleno-proteins (Ikram et al., 2021b). Due to these features, SeNPs are now being used in various agricultural and horticultural applications to mitigate biotic stress in plants (Zohra et al., 2021). Besides, SeNPs possess anti-bacterial activity and have potential to enhance plant physiological and biochemical properties (Ikram et al., 2022). SeNPs can enhance the activity of some enzymes and scavenge free radicals in plants, and they combine excellent bioavailability with low toxicity (Hashem et al., 2022). Furthermore, SeNPs are promising in stabilizing the immune system and activating the defense response of plants (Samynathan et al., 2023).

The exact mechanism of SeNP action is still unclear. However, some studies reported that the antimicrobial activity of plant-based SeNPs involves the generation of reactive oxygen species (ROS) and disruption of the integrity and stability of cell membranes (Filipović et al., 2021). Furthermore, plant based SeNPs can cause in pathogens breaking of cell walls, leakage of cellular contents and ATP. SeNPs can ultimately bind to the thiol groups of the membrane proteins and causes their loss of function, leading to cell death (Truong et al., 2021).

The current study was conducted to explore the impact of SeNPs from garlic clove extract on physio-biochemical properties, antioxidant defense system, fruit quality and protein regulation in huanglongbing infected ‘Kinnow’ mandarin plants. To the best of our knowledge this is the first such experimental study. It was hypothesized that SeNPs have the potential to enhance the antioxidant defense system and provide resistance against HLB causative agent, improving the quality of fruits from the diseased plants.

## 2 Materials and methods

### 2.1 Plant material

Seven-years-old ‘Kinnow’ mandarin plants, a hybrid of ‘King’ tangor (*Citrus nobilis*) × ‘Willow leaf’ mandarin (*Citrus deliciosa*) growing in loamy soil, were selected for the current study. All selected trees were showing HLB symptoms. The current research experiment was conducted in Tehsil Bhera, Village Melowal, Sargodha, Province of Punjab, Pakistan (latitude 32.447259° N and longitude 72.031278° E) during the winter of 2020. HLB-symptomatic plants were confirmed to be HLB-positive using PCR (Ikram et al., 2022). The ‘Kinnow’ mandarin plants selected for this study were separated from the other citrus plants using a red ribbon with a code number. The experiments were conducted in natural field conditions, and HLB-positive plants were normally irrigated at a three-week interval. All other horticultural procedures were performed according to the recommendation of the Pir Mehr Ali Shah Arid-Agriculture University, Punjab, Pakistan.

### 2.2 SeNPs preparation, application, and fruit sampling

Garlic extract was used for the green synthesis of SeNPs with 40-100 nm size, as described in our previous study (Ikram et al., 2022). Various SeNPs concentrations (25, 50, 75, and 100 mg L^–1^) were applied exogenously on the diseased plants, and garlic cloves extract (100 mL per plant) was also exogenously applied on HLB disease plants for comparison. SeNPs were applied using a sprayer machine (Hand Sprayer AP-20P, China). The solution was sprayed at 4:00 a.m. and 9 a.m. to ensure that the stomata were opening. Six treatments in three replicates were employed in a randomized complete block design (RCBD) (**Table 1**). Ten severely HLB-impacted branches on each treated plant were randomly selected and tagged. The branches were identified using yellow ribbons. All the treatments were applied two times with an interval of 15 days between the treatments on three occasions: before flowering (February 2020), at fruit setting (April 2020), and at the fruit growth stage (June 2020). For physio-chemical assessment, 21 fruits were collected from tagged branches for each treatment (seven fruits per plant). For proteomics analysis of the seeds, samples were collected from healthy plants or HLB-free (Control or T0 treatment), from untreated plants but HLB-infected (T1 treatment), and the HLB-infected plants treated with 75 mg L^–1^ (T5 treatment).

**Table 1:** Experimental layout of SeNPs application.

Treatments	Conditions
T0	Healthy plants (Control)
T1	HLB diseased plants (no treatment)
T2	HLB diseased plants + garlic extract
T3	HLB diseased plants + 25 mg L^-1^ SeNPs
T4	HLB diseased plants + 50 mg L^-1^ SeNPs
T5	HLB diseased plants + 75 mg L^-1^ SeNPs
T6	HLB diseased plants + 100 mg L^-1^ SeNPs

### 2.3 Assessment of physio-chemical parameters

#### 2.3.1 Measurement of fruit peel thickness, peel weight, fruit weight, fruit diameter, pH, and total soluble solids

Using a digital Vernier caliper, the thickness of the peel on both sides of the equatorially cut fruit and fruit diameter were measured in millimeters (mm). Electrical balance was used to weigh the fruit, peel, and rag contents in grams (g). The pH value was measured using a Hanna pH meter (Model HI 2211, Glassco Scientific & Analytical Company, Romania) that had been calibrated with pH buffer 7 and 4 calibration solutions. Total soluble solids (TSS) (°Brix) were measured using a portable refractometer (Model FG-103, Chincan, China) (Nawaz et al., 2019).

#### 2.3.2 Fruit juice extraction and quantification

Juice from fruit samples carefully harvested from all treatments was extracted using a standard electric fruit juicer and filtered (Nawaz et al., 2019). The following formula was used to calculate the percentage of fruit juice:


(1) $$Juice\;\left(\%\right)=\frac{Juice\;weight}{Fruit\;fresh\;weight}\times 100$$

#### 2.3.3 Measurement of titratable acidity

The titration method described earlier (Fabro et al., 2006; Nawaz et al., 2020b) was used with some slight alterations. First, 40 mL of distilled water (dH_2_O) was added to 10 mL of fruit juice. Then, by adding 4 g of NaOH anhydrous pellets to 1000 mL dH_2_O, sodium hydroxide (0.1N) was freshly made and used to titrate until a light pink hue appeared. To 10 mL of the juice solution, 2-3 drops of an indicator (phenolphthalein) were added for measurement. TA was determined as:


(2) $$TA\left(\%\right)=\frac{NaOH\;used\times NaOH\;normality\times 0.064}{Initial\;volume\;of\;juice\;used}\times 100$$

where 0.064 (mL^-1^) is the conversion factor.

#### 2.3.4 Ascorbic acid determination

Five mL of 1% HCl and 5 mL of juice were mixed, put in a new Eppendorf tube, and centrifuged for 10 min at 10,000 rpm. 0.3 mL of the mixture was collected after centrifugation, 2.7 mL of dH_2_O was added, and then absorption at 243 nm wavelength was measured using a spectrophotometer (Nawaz et al., 2019).


(3) $$\text{Ascorbic acid}\;\left(\text{arb}.\text{ units}\right)=\left(\frac{\text{ absorbance of sample taken at }243\text{ nm}}{0.293}\right)\times 10,$$

where 0.293 represents the specific molar absorpitivity of ascorbic acid at 243 nm.

### 2.4 Assessment of biochemical parameters of fruit

#### 2.4.1 Estimation of reducing sugar contents in fruit juice

Fruit sugar content was measured using the standard method (Nawaz et al., 2020b) with slight alterations. 10 mL potassium oxalate (7%) and 25 mL lead acetate (2%) were added to a 250 mL beaker containing 10 mL juice and filled the beaker with distilled water to get volume of 250 mL. 10 mL of Fehling solution (5 mL of each Fehling A and B) were used to test the sample. 69.3 g of copper sulfate pentahydrate was dissolved in 1000 mL pf distilled water to prepare Fehling A, while 100 g of sodium hydroxide anhydrous and 345 g of sodium potassium tartrate in 1000 mL distilled water to get Fehling B. The mixture was heated until red precipitates appeared.


(4) $$\text{Reducing sugars}\left(\text{g}/100\text{ mL}\right)=\frac{6.25\left(\text{X}\right)}{\text{Y}}$$

where X - the amount of standard sugar (g), Y - sample volume used (in 100 mL), and 6.25 – the empirical coefficient obtained from the standard curve (Nawaz et al., 2020a).

#### 2.4.2 Estimation of total sugars in fruit samples

The previously used method (Nawaz et al., 2020b) was used with slight alterations.

Briefly, 25 mL of sample used for reducing sugar was put in a 50 mL beaker followed by addition of 5 mL concentrated HCL and 20 mL of distilled water. The mixture was kept in dark overnight. After that 2-3 drops of phenolphthalein indicator and NaOH were added to the solution and titrated it with Fehling solution.


(5) $$\text{Total sugars}\left(\text{g}/100\text{ mL}\right)=\frac{25\left(\text{X}\right)}{\text{Y}}$$

where X - the amount of standard sugar (g), Y - sample volume used (in 100 mL), and 25 - the empirical coefficient obtained from the standard curve (Nawaz et al., 2020b).

#### 2.4.3 Estimation of non-reducing sugars in fruit samples

The concentration of non-reducing sugars was computed from that of the total sugars and reducing sugars following the methodology and formula of (Hortwitz, 1960):


(6) Non−reducing sugars (g/100 mL)=total sugars –reducing sugars×0.95,

where 0.95 is a correction factor (Hortwitz, 1960).

#### 2.4.4 Determination of antioxidant enzymes in citrus fruit juice

To determine catalase (CAT; EC 1.11.1.6) in fruit juice, 0.5 mL of fresh juice was homogenized with 5 mL of 50 mM phosphate buffer (pH 7.0) and 2% H_2_O_2_. Then the samples were centrifuged at 4°C for 10 min at 1200 rpm. The spectrophotometer (Shimadzu, model UV 1800, Kyoto, Japan) was used to record the catalase activity at an absorbance of 240 nm for 2 min (Aebi, 1984). The activity of superoxide dismutase (SOD; EC 1.15.1.1) was evaluated according to the methods of (Beauchamp and Fridovich, 1971; Giannopolitis and Ries, 1977) with slight modifications by assessing catalase’s ability to prevent the photochemical reduction of nitro blue tetrazolium (NBT). The absorbance of SOD activity was measured at 560 nm using a spectrophotometer. The peroxidase (POD) activity was assessed by 470 nm absorbance using the methods of (Chance and Maehly, 1955) with slight modifications.

#### 2.4.5 Determination of flavonoids contents

The TFC contents were measured following the protocol of (Nongalleima et al., 2017). Plant juice extract was prepared using pure ethanol (1:10). 1 mL of plant extract was put in a glass cuvette followed by the addition of 10% aluminium chloride, 10% sodium acetate and 1.2 mL of distilled water. The sample was kept in dark for 2 h and the absorbance was measured at 415 nm by a spectrophotometer. For control, 0.1 mL of blank sample was prepared containing only aluminium chloride solution. Another blank sample (0.1 mL AlCl_3_ + 0.1 mL sodium acetate and 1.3 mL of distilled water) was prepared without plant extract. Standard curve was measured using quercetin.


(7) TFC (mg of Quer. equal/100 mL or 100 g)=(Sample absorbance + 0.01)/0.024,

where 0.01 is the absorbance correction factor and 0.024 is the calibration factor derived from a standard curve of quercetin.

#### 2.4.6 Total phenolic contents

One mL of fresh juice from samples was diluted up to the volume of 25 mL. The samples were centrifuged at 15,000 rpm for 25 min at 4°C. TPC contents were measured in supernatant by an UV-visible spectrophotometer, and gallic acid was used as a standard. The methods of (Miliauskas et al., 2004) and (Singleton and Rossi, 1965) were deployed with slight modifications. Briefly, 0.2 mL of the sample extract was put into tubes containing 1.0 mL of Folin-Ciocalteu’s reagent diluted in H_2_O. After 12 min incubation, 0.8 mL of a sodium carbonate solution (7.5% w/v) was added to the sample. The tubes were kept at room temperature (RT) for 35 min and absorbance at 743 nm was measured. The TPC was expressed as gallic acid equivalents (GAE) in mg/100 mL of fruit juice:


(8) TPC (mg of GAE/100 mL or 100 g)=(Absorbance value of sample+0.058)/0.102

The constants 0.102 and 0.058 represent values derived from the calibration curve.

### 2.5 Proteome analysis of ‘Kinnow’ mandarin seed samples

#### 2.5.1 Protein extraction from the seeds

The seeds were extracted from ‘Kinnow’ fruit with three treatments (T0, T1, and T5), and nucellus halves were separated with the help of a scalpel (Satti et al., 2022; Sohail et al., 2022). The nucellus tissues (400 mg) were weighed and placed in 50 mL Falcon tubes to which a small amount of liquid nitrogen was added. After that, the seeds were put into pre-cooled mill jars, then into the MM400-Mill (Retsch), and ground for 1 min at 30 Hz. The mill jars were once more submerged in liquid nitrogen after grinding to make the powdered nucellus tissues brittle. The powder was then placed in a mortar and ground for 10 s before adding the lysis buffer to the mill jars for another 2 min. The protein-containing supernatant was then removed from the extract by centrifuging at 14,000 rpm for 10 min at 4°C. To get rid of all debris, the centrifugation process was repeated. After that, 400 µL of the supernatant was quickly placed into Eppendorf tubes to which four times the pre-chilled 100% acetone volume was added and vortexed. Following that, the solution mixtures were centrifuged for 10 min at 4°C at 14,000 rpm. The supernatant was treated for 1 h at -20°C to precipitate protein and then removed. The protein pellet was re-suspended in 100 µL of urea buffer and sonicated using the probe for 60 s at 20% amplitude to ensure that all protein was dissolved. Bicinchoninic Acid Assay (BCA) was used to measure protein concentration using as a standard bovine serum albumin (BSA) (Walker, 2009).

#### 2.5.2 Protein digestion for mass spectrometry analysis

Dithiothreitol (DDT) 0.5 M was used to reduce sample’s proteins (100 mg) for 30 min at 37°C, and the free cysteine residues in the proteins were then alkylated with 0.5 M iodoacetamide (IAM) for an hour at RT in the dark. Trypsin was used to digest the alkylated proteins at a ratio of 1:100 (enzyme to protein) for 16 h at 37°C. Formic acid (FA) was used to terminate the reaction. After being cleaned and desalted (**Table 2**), the acquired samples were concentrated by a SpeedVac and subjected to a nanoLC-MS/MS analysis.

**Table 2:** Cleaning and desalting of peptides.

Peptide cleaning/desalting using C18 SepPac			(CV = 150 µL)
Initialize	3CV	1 x 450 µL	MeOH
Wash TFA	3CV	1 x 450 µL	50% ACN/0.1% TFA
Equilibrate	6CV	1 x 900 µL	0.1% TFA
Sample load (pH 1-3)	≥1CV	≥1 x 150 µL	in 0.1% TFA
Wash/desalt 1	3CV	1 x 450 µL	0.1% TFA/2% ACN
Wash/desalt 2	3CV	1 x 450 µL	0.1% FA/ 2% ACN
Elution 1	3CV	1 x 150 µL	30% ACN/0.1% FA
Elution 2	2CV	1 x 300 µL	80% ACN/0.1% FA

CV, Column volumes; MeOH, Methanol; TFA, Trifluoroacetic Acid; ACN, Acetonitrile; FA, Formic Acid.

#### 2.5.3 Nanoflow-liquid chromatography-tandem mass spectrometry analysis

Peptides were placed into a Dionex 3000 RPLC nanoflow system with a C18 PepMap trap column. With a 300 nL/min flow rate and a 2 kV spray voltage, the peptides were eluted from the trap column using 0.1% FA in ACN. The Orbitrap Q Exactive mass spectrometer (Thermo Fisher Scientific) analyzed peptides in data-dependent acquisition mode. Full mass spectra covering the m/z range 375-2000 were recorded at a nominal resolution of 60,000. A lock mass function (m/z 445.120025) was applied for high mass precision. The following values were used for the ion isolation window: the activation type was high collision-induced dissociation (HCD), the activation time was 30 ms, the minimum required signal was 5000, isolation width 2 m/z, the default charge state of 2+, and the normalized collision energy of 35%. Dynamic exclusion were as follows: exclusion list size was set at 500, exclusion mass width at 1.5 Da, exclusion duration at 60 s, the repetition period was 30 s, and the number of repetitions was 1.

#### 2.5.4 Identification of peptides from mass spectrometry data

The Mascot search engine (v. 2.5.1, Matrix Sciences, Londin, UK) was used to identify the peptides in the UniProt Knowledgebase SwissProt database (http://www.uniprot.org, assessed on 27 September 2022) with *Citrus reticulata* L. selected for protein identification. The search parameters were as follows: methionine oxidation was a variable modification, whereas cysteine’s carbamidomethylation was a fixed modification. The trypsin was designated as an enzyme of protein digestion, and one missed cleavage was accepted. Peptide mass tolerance was considered 0.8 Da and MS/MS fragment mass tolerance - 10 ppm. The Mascot search results were filtered to keep the false discovery rate (FDR) below 1%. To normalize the protein abundances, each protein’s abundance was multiplied by a million and divided by the total of all protein abundances in a given replicate.

### 2.6 Statistical analysis

The protein abundances were statistically evaluated using the Statistical Package for Social Sciences (SPSS). When just two groups were compared, significance was determined using a basic Student’s t-test. Proteins differing abundances between the control and treated groups at P < 0.05 were found (Satti et al., 2022).

### 2.7 Bioinformatics analysis

The Uniprot website (http://www.uniprot.org) was used to analyze the functions of each up and down-regulated protein and metabolic pathways, and functional enrichment was assessed through STRING, a search tool for retrieval of interacting proteins/genes, (https://string-db.org).

## 3 Results

### 3.1 Impact of garlic extract mediated SeNPs on physio-chemical profiling of HLB infected citrus plants

**Figure 1:**
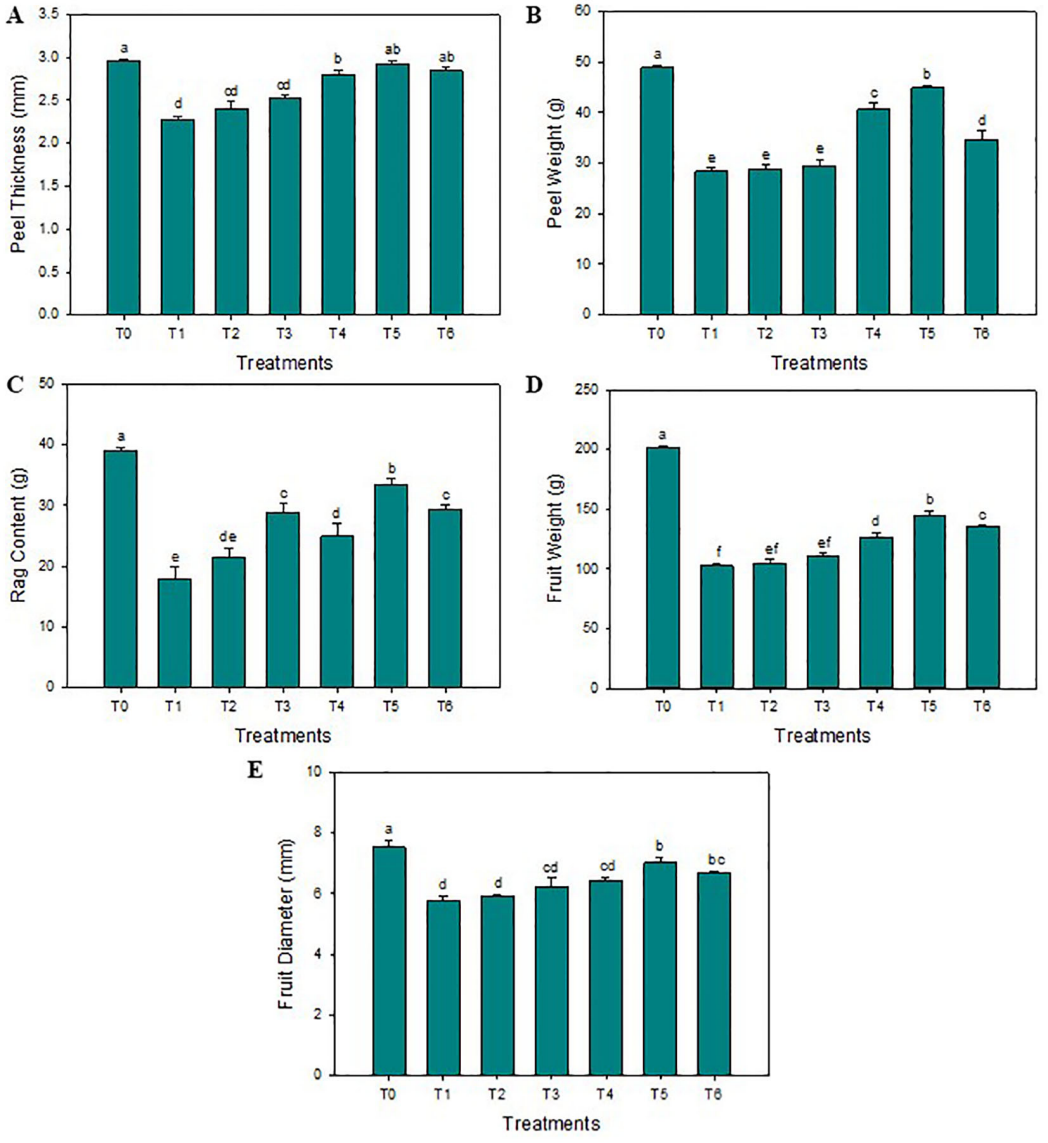
Impact of SeNPs on **(A)** Peel thickness, **(B)** Peel weight, **(C)** Rag content, **(D)** Fruit weight, and **(E)** Fruit diameter. Bars with the same lettering are not significantly different. Error bars indicate the Standard Deviation (SD).

**Figure 2:**
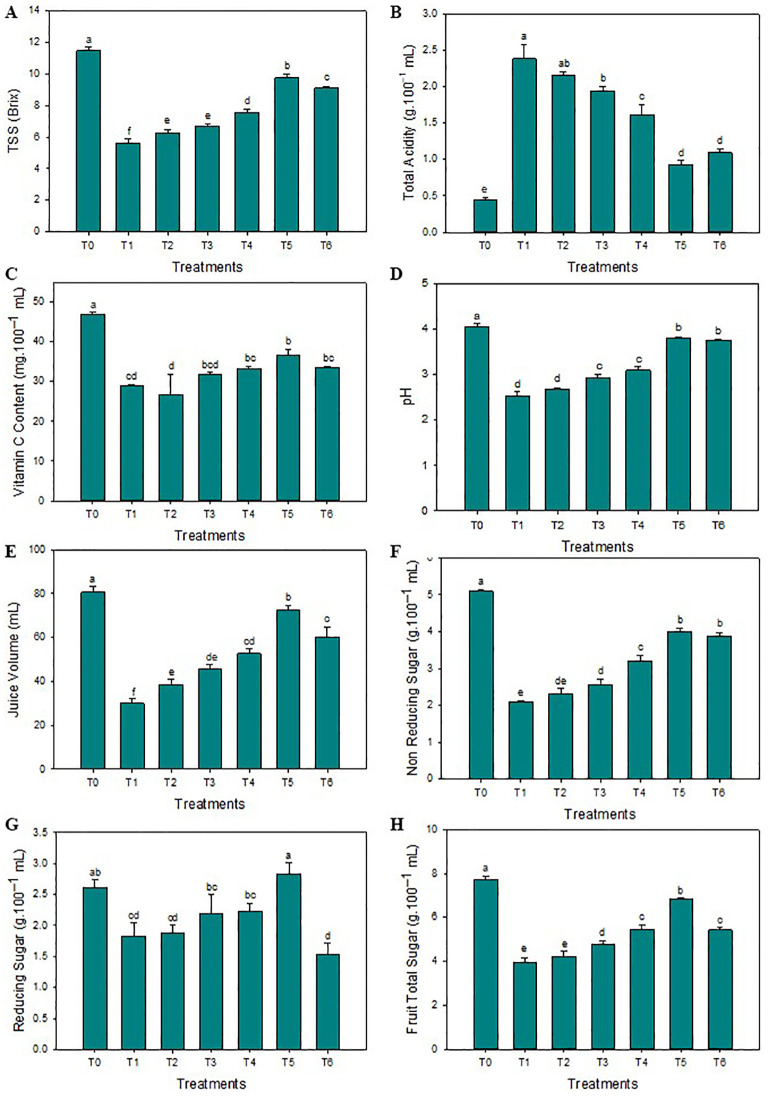
Impact of SeNPs on **(A)** Peel TSS, **(B)** TA content, **(C)** Vitamin C content, **(D)** pH, **(E)** Juice volume, **(F)** Non-reducing sugar, **(H)** Reducing sugar, and **(G)** Total sugar. Bars with the same lettering are not significantly different. Error bars indicate the Standard Deviation (SD).

The SeNPs have effectively enhanced the thickness of the peel compared to untreated diseased plants. The highest average peel thickness was reported when the 75 mg L^-1^ concentration of SeNPs was applied on HLB-infected citrus plants (3.3 mm), and minimum peel thickness was noticed in untreated diseased plants (2.2 mm). Furthermore, compared to untreated diseased plants, the maximum average peel and rag contents of 29.1% and 24.1% were noticed in response to 75 mg·L^–1^ of green SeNPs. Similarly, the average fruit weight was significantly enhanced compared to control plants (144.7 g), and the minimum average fruit weight was observed in untreated diseased plants (104.8 g). Unexpectedly, the average fruit weight decreases to 140.1 g when the SeNPs concentration rose to 100 mg L^–1^. The maximum fruit diameter was observed in healthy plants (7.5 cm), but the minimum fruit diameter was observed in untreated diseased plants (5.8 cm). SeNPs at the 75 mg·L^–1^ concentration were most efficient in enhancing the diameter of diseased fruit (7.1 mm) in contrast to untreated diseased plants, see **Figure 1**. Similarly, the maximum total soluble solids (TSS) were observed in healthy citrus fruits (11.5°Brix), while the minimum amount of TSS was noticed in untreated diseased plants (5.6°Brix). An exogenous spray of SeNPs on citrus fruits with HLB disease considerably and dose-dependently enhanced the TSS contents. The maximum contents of TSS (9.6°Brix) in HLB-infected citrus plants were observed at the 75 mg L^-1^ SeNPs concentration. Moreover, the highest titratable acidity (TA) was noticed in diseased citrus fruits at about (2.4 g/100 mL), but a minimum TA concentration was determined in healthy citrus fruits (0.45 g/100 mL). Interestingly, TA contents decreased in HLB-infected plants (0.92 g/100 mL) treated with SeNPs at the concentration of 75 mg L^-1^. Unfortunately, the level of TA increased at the 100 mg L^-1^ concentration of SeNPs. Additionally, maximum ascorbic acid was noted in healthy citrus fruit (46.2 mg/100 mL of juice) while minimum (29.8 mg/100 mL of juice) was observed in untreated HLB-infected citrus plants. Ascorbic acid content (37.2 mg/100 mL of fruit juice) was enhanced in HLB-infected citrus fruit treated with SeNPs at 75 mg L^-1^ in comparison with untreated HLB infected fruits. The maximum fruit juice’s pH (4.04) and juice volume (80.4 mL) was determined in healthy plants. In untreated diseased fruit juice, a minimum pH (2.53) and average juice content (29.2 mL) was found. Compared to that, in HLB diseased citrus fruits under the foliar application of 75 mg L^-1^ SeNPs, both the pH and volume were found elevated (3.73 and 71.1 mL, respectively) compared to untreated diseased fruits, see **Figure 2**.

### 3.2 Impact of garlic extract mediated SeNPs on biochemical properties of HLB infected citrus plants

**Figure 3:**
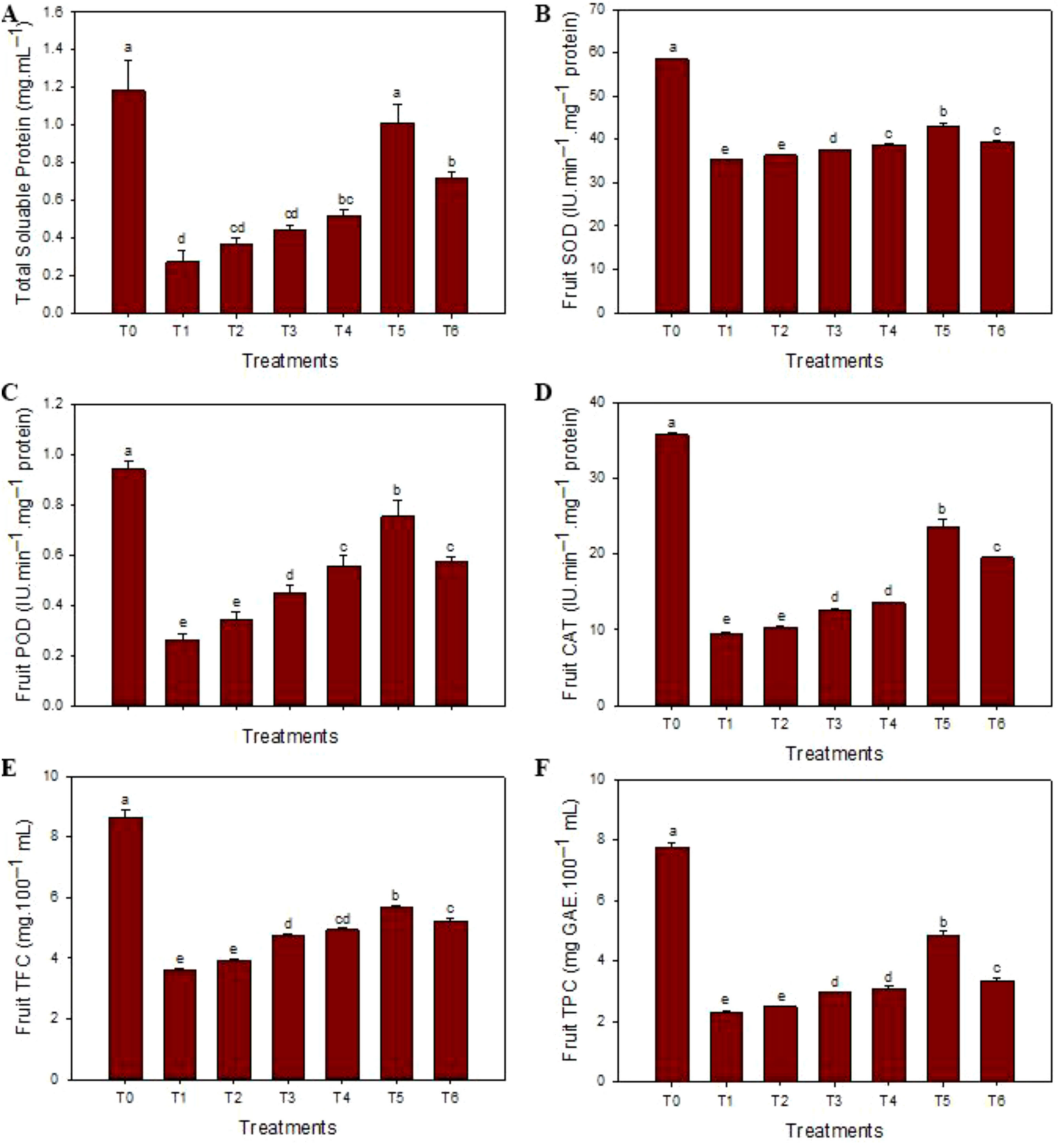
Impact of SeNPs on **(A)** total protein content, **(B)** superoxide dismutase (SOD) content, **(C)** peroxidase (POD) content, **(D)** catalase (CAT) content, **(E)** flavonoids (TFC) content, and **(F)** phenolic (TPC) content. Bars with the same lettering are not significantly different. Error bars indicate the Standard Deviation (SD).

The highest contents of total soluble proteins (1.15 mg·mL^–1^) were observed in healthy fruit juices, and the minimum contents (0.33 mg·mL^–1^) were noticed in HLB-infected ‘Kinnow’ mandarin fruit juices. SeNPs at 75 mg L^–1^ enhanced the total soluble protein contents (1.01 mg·mL^–1^). Similarly, maxima of antioxidant enzyme SOD (58.3 IU min^–1^ mg protein^–1^), POD (0.95 IU min^–1^ mg protein^–1^), and CAT (35.1 IU min^–1^ mg protein^–1^) contents were observed in healthy fruits while the minimal concentrations (33.1 IU min^–1^ mg protein^–1^, 0.54 IU min^–1^ mg protein^–1^, and 23.1 IU min^–1^ mg protein^–1^, respectively) were noticed in diseased plants without any treatment. The maximum contents of SOD (41.9 IU min^–1^ mg protein^–1^), POD (0.75 IU min^–1^ mg protein^–1^), and CAT (19.2 IU min^–1^ mg protein^–1^) were determined in the HLB-infected ‘Kinnow’ mandarin fruit juices when 75 mg L^–1^ concentration of SeNPs was exogenously applied. Similarly, the maximum level of TPC (7.5 mg GAE.100 mL^–1^) and TFC (8.6 mg of quer. Equal 100 mL^–1^) contents were observed in healthy fruit juice, and minimum contents of TFC (3.62 mg of quer. Equal/100 mL) and TPC (2.15 mg GAE.100 mL^–1^) and were determined in diseased untreated ‘Kinnow’ mandarin fruit juices, respectively. Moreover, 75 mg L^–1^ concentration of SeNPs maximum increased the level of non-enzymatic TPC (4.52 mg GAE.100 mL^–1^) and TFC (5.16 mg of quer. Equal 100 mL^–1^) contents in HLB affected ‘Kinnow’ mandarin fruit juices. Similarly, maximum reducing sugar (2.61 g·100 mL^–1^), non-reducing sugar (5.12 g·100 mL^–1^), and total sugar content (7.70 g·100 mL^–1^) were noted in healthy citrus plants, while minimum concentration was noted in untreated diseased ‘Kinnow’ mandarin fruits. The SeNPs at 75 mg L^–1^ enhanced the maximum sugar content level compared to untreated diseased ‘Kinnow’ mandarin fruits (**Figure 3**).

**Figure 4:**
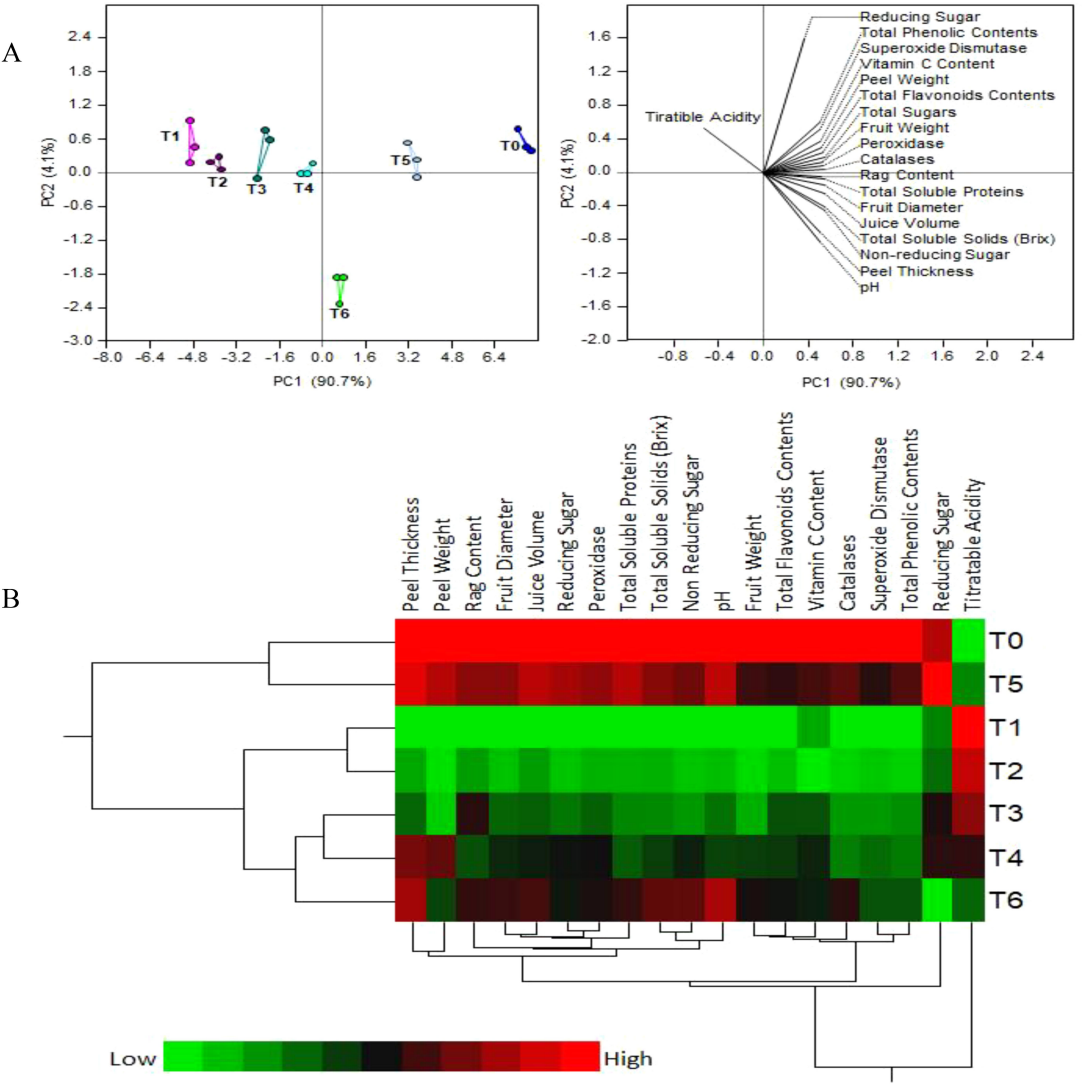
**(A)** Principal component analysis (PCA) and **(B)** two-way hierarchical cluster analysis (HCA) of the physio-chemical and biochemical parameters of fruit juice from HLB-infected ‘Kinnow’ mandarin fruit treated with various concentrations of Se-NPs.

**Figure 4** shows the results of principal component analysis and two-way hierarchical cluster analysis of the physico-chemical and biochemical parameters of fruit juice. The first two PCA components contributed more than 95% of the variance among the treatments: PC1 - about 90.7% and PC2 – around 4.1% (**Figure 4A**). A strong separation between the SeNPs treatments is observed, mostly along PC1, with replicates of each treatment grouping together and separately from other treatments. As expected, T1 (diseased plants with no treatment) and T0 (control healthy plants) are the most separated groups, while T5 (HLB diseased plants + 75 mg L^-1^ SeNPs) is the closest to healthy control.

The heat map of two-way hierarchical cluster analysis (HCA) on **Figure 4B** is colored according to numerical values, with low values being green and high values - red. This heat map agrees largely with the PCA results. Briefly, there are three distinct clusters: group 1 (T0 & T5), group 2 (T1 &T2), and group 3 (T3, T4 & T6). However, unlike PCA, HCA put T1 close to T5, while the color bars of these treatments are clearly very different. Therefore, PCA is the preferred method of data analysis, whole HCA results must be taken with some caution.

### 3.3 Proteins expression analysis of SeNPs treated HLB-infected ‘Kinnow’ mandarin plant fruits

The proteins expression analysis study was performed to examine the effect of SeNPs synthesized from garlic cloves extract on seeds from HLB-infected ‘Kinnow’ mandarin plants. The analysis was carried out at Karolinska Institutet, Stockholm (Sweden). Fruit and seed samples were taken from three treatments: T0, T1, and T5. In a pairwise comparison of the T1 (untreated disease) and T0 (healthy control), 182 and 186 proteins were found in each sample, respectively, with 133 common proteins. Of these latter, 75 proteins were significantly upregulated under HLB stress, and 58 proteins were down-regulated (see the full list of proteins in **Supplementary Table 3**). For instance, proteins associated with glutamate 5-kinase activity and cysteine-type peptidase activity show significant upregulation, while those linked to glutathione transferase activity and phosphoprotein phosphatase exhibit marked downregulation. In a comparison of T0 and T5 treatments, 211 proteins and 199 proteins were identified, respectively, with 143 common proteins. Of these, 83 proteins showed up-regulation and 60 - downregulation. In T5 (HLB diseased plants treated with 75 mg L^-1^ SeNPs), proteins associated with translation elongation factor activity, peroxidase activity, superoxide dismutase activity and structural constituents of ribosomes associated proteins are significantly upregulated compared to the healthy control T0. On the other hand, downregulated proteins include aspartic-type endopeptidase and metalloendopeptidase.

#### 3.3.1 Functional categorization of up and down-regulated proteins in the seeds of ‘Kinnow’ mandarin in response to biotic stress of HLB disease treated with 75 mg·L^–1^ concentration of SeNPs

**Figure 5:**
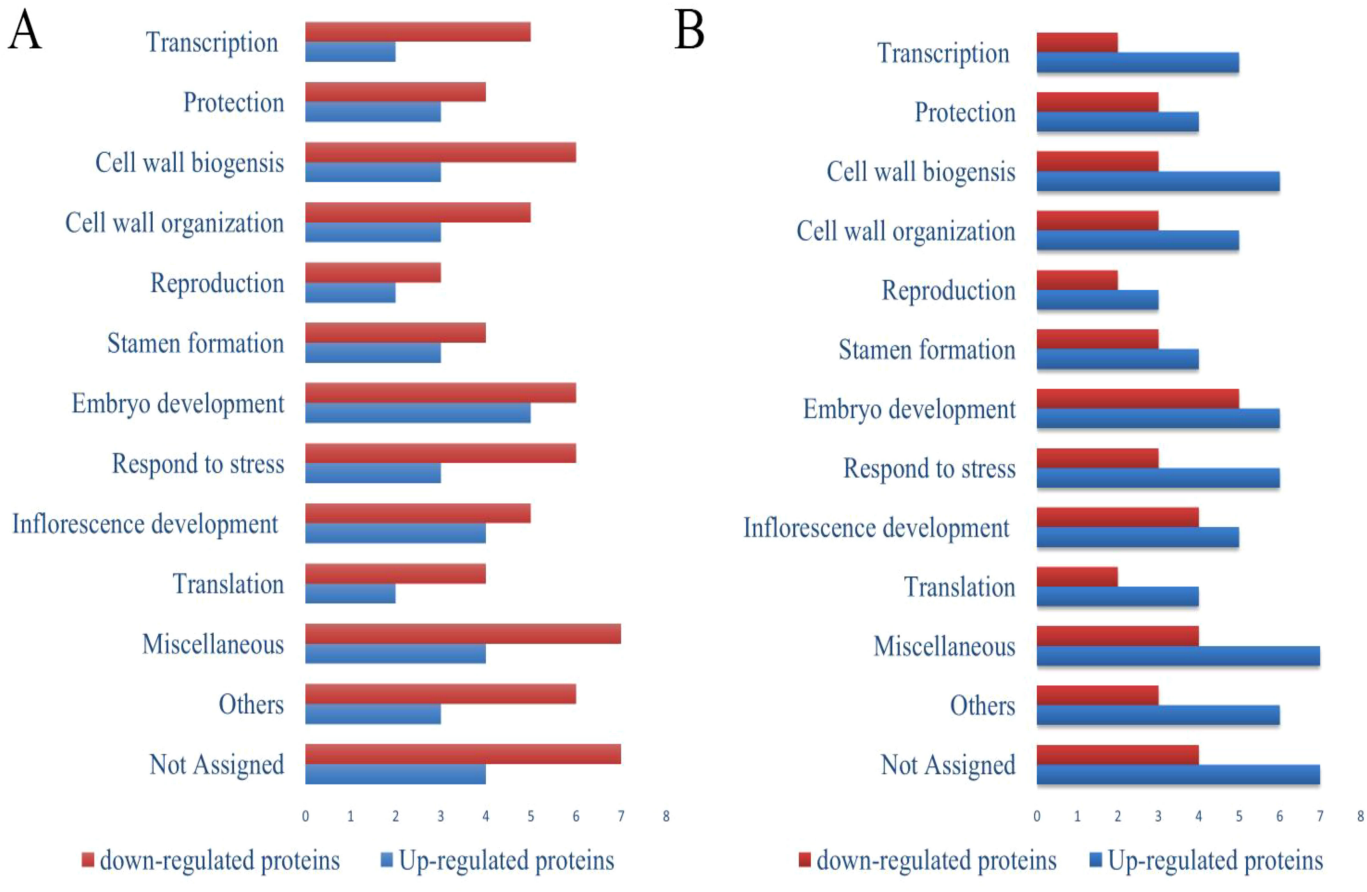
Functional categorization of proteins in seeds of ‘Kinnow’ plants up- and down-regulated compared to healthy controls (T0). **(A)** untreated disease plants (T1), and **(B)** diseased plants treated with 75 mg L^–1^ of SeNPs (T5). The full list of proteins with significant abundance changes (p<0.05) is given in **Supplementary Table 3**. Blue and red columns mean up-regulated and down-regulated proteins, respectively.

Pathway analysis of the proteomics results revealed that the downregulated proteins related to transcription, protein biosynthesis, protection, translation, RNA processing, DNA binding, protein refolding, reproduction, inflorescence development, stamen formation, and cell redox homeostasis under biotic stress. At the same time, the upregulated proteins are related to folic acid metabolism, glucose metabolic processes, and metalloendopeptidase activity. Foliar applications of 75 mg·L^–1^ concentrations of SeNPs significantly upregulated the proteins related to transcription, cell wall biogenesis, embryo development, ATP binding, glutathione transferase activity, metal ion binding, translation, response to the pathogenic attack, pyruvate dehydrogenase activity, hydrogen peroxide removal, pollen tube formation, reproduction inflorescence development protection, cell wall organization, translation and detoxification, and reproduction (**Figure 5**). Furthermore, our results revealed that HLB disease significantly altered the proteomics expression profile in seed samples. Many proteins associated with translation, inflorescence development, embryo development, stamen formation, protein transport, reproduction, and cell wall organization were downregulated. SeNPs improved protein expression by upregulating the proteins associated with RNA processing, lipid metabolism, cell wall modifications, peroxidase activity, translation elongation activity etc.

## 4 Discussion

### 4.1 Impact of garlic extract mediated SeNPs on physio-biochemical attributes of HLB infected “Kinnow” mandarin plants

The present results show that, in general, SeNPs have positive impact on the physio-chemical and biochemical properties of the fruits from HLB-infected citrus plants. Upon treatment, such desired parameters as fruit diameter, fruit weight, total soluble solids, etc., have increased, while the undesired parameters, such as the titratible acidity in fruit juice, have reduced compared to untreated control. Similar results were also obtained in an earlier study (Zahedi et al., 2019a), where SeNPs have enhanced the fruit’s physical and biochemical attributes in pomegranate plants subjected to stress. Another study reported that SeNPs at the concentration of 25 mg L^-1^ have increased the firmness of fruit and TSS in *Cucumis sativus* (Shalaby et al., 2021). In *Solanum lycopersicum*, the foliar application of 100 mg L^-1^ SeNPs caused an increase in TSS compared to untreated plants (Saffan et al., 2022). A trend of increase in TSS and lowering of TA was noticed in *Punica granatum* fruiting species treated with SeNPs at the concentration of 2 µM (Zahedi et al., 2019a). The betterment in fruit physio-chemical attributes may be due the fact that Se acts as a catalytic center in selenoproteins essential in redox pathways. Selenium is also involved in the antioxidant defense system involving glutathione peroxidase enzymes, which scavenge ROS and protect the physiology of plants (Gupta and Gupta, 2017). In that work, it was also observed that a high concentration of SeNPs (100 mg L^-1^) has reduced the TSS contents and increased the TA in fruit samples, possibly due to selenosis caused by excess of Se. It is also reported that high doses of Se induce oxidative stress in plants, which in turn damages their morpho-physiological and biochemical parameters (Ahmad et al., 2023). Concludingly, SeNPs have a positive impact on the fruit physio-chemical profile, but require choosing optimal concentration.

Plants evolve strong antioxidant defense systems, including enzymatic and non-enzymatic components, to prevent the damaging consequences of oxidative stress under biotic and abiotic stress conditions (Şen, 2012). Antioxidant enzymes include the most effective intracellular metalloenzymes like SOD, CAT, and POD (Berwal et al., 2021). Plants respond to drastic effects of oxidative damage by changing the expression level of enzymes in antioxidant defense (Al-Wasfy, 2013). The low production of superoxide dismutase enzymes in untreated diseased plants reveals that the immune response of mandarin plants is by itself too weak to defend against microbial infection. In our experiments, SeNPs showed a dose-dependent response up to optimal concentration of 75 mg L^–1^, and they enhanced the enzymes related to antioxidant defense in HLB-infected plants. However, these enzyme contents were reduced at a high SeNPs level of 100 mg L^–1^. This may be due to the excess of selenium, which might cause a nutrient imbalance (Ikram et al., 2021a).

A parallel to our findings can be drawn from a research study describing oxidative damage in strawberry plants as indicated by high levels of hydrogen peroxide and malondialdehyde contents in plant leaves (Zahedi, Abdelrahman, et al., 2019). In that study, foliar applications of SeNPs elevated the performance of enzymes related to antioxidant defense and reduced the amount of H_2_O_2_ and MDA contents. Similar results were obtained in other plant species (Zahedi et al., 2019b). Our findings that the SeNPs enhanced the activities of enzymes in ‘Kinnow’ mandarin fruits under biotic stress are similar to the results of (Jiang et al., 2017), where SeNPs altered the gene expression and, ultimately, enhanced the performance of antioxidant defense-associated enzymes in maize plants. Another study showed that SeNPs improved the enzyme content in lettuce and melon plants under environmental stresses, while eliciting resistance against tomato late blight disease by modulating defense gene expression (Berwal et al., 2021). The same study reported noteworthy up-regulation of antioxidant defense-associated enzymes by SeNPs application during the biotic stress of late blight disease. In some other studies, SeNPs showed upregulation of SOD, CAT, POD enzymes under stress conditions (Handa et al., 2019; Joshi et al., 2021). One other study reported the positive effects of SeNPs on the flavor quality and fruit development of the tomato plants. SeNPs promoted the ascorbic acid-glutathione cycle and precursors of antioxidants to resist against the oxidative stress by increasing the level of phytohormones helping the maturation of tomato fruits (Liu R. et al., 2024). Furthermore, SeNPs restored the fruit quality by regulating the expression of stress associated genes to enhance the level of fructose, glucose and volatile organic compounds. Our results are also in line with the earlier study where biologically prepared SeNPs dramatically increased the antioxidant capability of sesame plants under biotic stress by enhancing the level of SOD enzymes by 147%, POD by 140% and CAT by 76% (Ahmad et al., 2023).

Antioxidant enzymes carry out the conversion of H_2_O_2_ into H_2_O and O_2_ and reduce oxidative stress in the plants. Biotic stresses causes overproduction of ROS, while the balance between their production and scavenging determines the cell fate (Gratão et al., 2005). The exact antioxidant defense mechanisms of suppressing HLB causative agents by SeNPs is still unclear and debatable. Among the mechanisms reported to date are the impaired membrane function (loss of membrane potential, membrane damage), protein dysfunctioning and dysregulatiom of the nutrient uptake (Sultana et al., 2023). It is known that NPs attach to microbial cell membrane due to electrostatic attraction to negatively charged plasma membrane of bacteria. The attached NPs cause disruption in membrane structure and stability. This, in turn, causes leakage of internal cell content including enzymes, proteins and metabolites, and results in cell death (Ikram et al., 2021a).

Moreover, Se acts as a stimulant inducing stress-associated antioxidant defense systems (Das et al., 2018). Flavonoids and phenolic compounds are antioxidants that produce many important secondary metabolites and perform cellular signaling under stress conditions, shielding plants from severe oxidative damage (Liu et al., 2022). Various research studies (Michalak, 2006; Handa et al., 2016) have suggested that NPs are beneficial in producing antioxidant compounds in plants, which supports our results. Remarkably, the effect of Se increased when it was applied in the form of NPs (González-Morales et al., 2017; Ruotolo et al., 2018). Zahedi et al. have found that SeNPs enhanced TPC and TFC in pomegranate fruits under stress conditions (Zahedi et al., 2019a). In another study, Se upregulated the biosynthesis of phenyl-propanoid compounds, followed by a buildup of flavonoids and polyphenols in *Fragaria×ananassa* (Mimmo et al., 2017).

Our results also showed that SeNPs enhance the total soluble sugar contents in the fruits of HLB-infected plants. It has been noted that an increase in soluble sugars could lead to a rise in the total soluble solids (Morales-Espinoza et al., 2019). The soluble solids in citrus fruits have great importance because of the effect on their organoleptic properties (in simple terms, fruits taste better). Furthermore, the fruit quality is strongly affected by the pH of fruit juice. A previous study also reported that HLB-infected fruits had higher acidity, lower sugars, and vitamin C and enhanced bitterness (Bové, 2006). As less acidic fruits are more appreciated by consumers, applying SeNPs to disease plants improves their market potential. Enhancement in the total soluble solid’s content in response to various concentrations of SeNPs was reported for potato plants (Ibrahim and Ibrahim, 2016), as well as alfalfa (Owusu-Sekyere et al., 2013), where it was associated with increased activity of fructose 1,6-bisphosphatase.

There is less data available for the use of SeNPs in ‘Kinnow’ mandarin fruits under stress conditions. However, some other studies showed that foliar application of extracts containing Zn and B augmented the total sugar content in citrus fruits (Babu and Yadav, 2005), with favorable impact on fruit flavor. Another previous study reported that Se/Si nanoparticles enhanced the total phenolic, vitamin C, and antioxidant capacity in strawberry fruits (Zahedi et al., 2020), supporting our findings. Yet another study also reported the that *Nigella sativa* mediated SeNPs positively influence the growth, yield and biochemical parameters of tomato plants under stress. SeNPs increased the fruit yield (> 100%) and fruit weight (> 100%), while lycopene content was also increased by 75%. Ascorbic acid content, proteins, phenolic and flavonoids were also enhanced by using the NPs in tomato plants under stressed environment (Ahmad et al., 2024).

### 4.2 Impact of garlic extract mediated SeNPs on proteomics expression of HLB infected “Kinnow” mandarin plants

Proteomics helps to understand the role and mechanisms of NPs in plants by correlating the metabolic processes in cells with gene expression (Driessen et al., 2015). To the best of our knowledge, this is the first study to explore at the proteome level the role of SeNPs in managing HLB-infected citrus plants. During the attack of a pathogen, a variety of defense-responsive proteins are released by the host plant in the apoplast to impede the perceived pathogenic attack (Ruano and Scheuring, 2020). Our proteomics results revealed that the glutamate 5-kinase activity and cysteine-type peptides activity-associated proteins were significantly up-regulated in untreated HLB-diseased ‘Kinnow’ mandarin fruit. Glutamate 5-kinase activity associated proteins are involved in proline biosynthesis pathways and their activity plays a significant role in plant defense under environmental stresses (Pavlíková et al., 2008). The upregulation of cysteine-type peptidase activity associated proteins in the current study may be due to the *C*Las infestations of the mandarin plants (as mentioned above, *C*Las are among the species causing the HLB symptoms). Cysteine-type peptidase plays a key role in the defense mechanisms of plants under biotic stress. These proteins are involved in the proteolysis process, in which they degrade the cellular proteins into peptides that provide signals for defense under biotic stress (Liu Y. et al., 2024). In addition, these proteins modulate some hormone-associated pathways that regulate plant defense responses. So, it could be speculated that the upregulation of these proteins acts as a first line of defense against the biotic stress of HLB.

Among the proteins significantly down-regulated in untreated diseased plants are those associated with the activity of oxidoreductase, glutathione transferase, and superoxide dismutase (**Supplementary Table 3**). These proteins are involved in cell redox homeostasis, stress response and protection against oxidative stress (Bose et al., 1999; Graifer and Karpova, 2015; Gucciardo et al., 2007). Similar to the current study, the proteins associated with the oxidoreductase activity also decreased in abundance in maize plants under biotic stress (Hafiz et al., 2022). The down-regulation of the above proteins may be due to the overproduction of H_2_O_2_ in infected mandarin fruits, similar to the excessive production of H_2_O_2_ in *C*Las infected ‘Valencia’ sweet orange (*Citrus sinensis*) (Ma et al., 2021). In that study, *C*Las infection downregulated the antioxidant defense system associated genes, supporting that HLB causes oxidative stress.

Normally, H_2_O_2_ in healthy plant is detoxified by peroxidase enzymes that convert H_2_O_2_ into H_2_O and O_2_ (Pandey et al., 2017). Therefore, excess of H_2_O_2_ in diseased plants could be caused by the deficit of peroxidases, which is consistent with the observed down-regulation of peroxidase enzymes in untreated infected plants. On the other hand, the proteomics analysis in our study revealed a significant upregulation of peroxidase-associated proteins in response to SeNPs treatment infected fruits, consistent with the observed increased peroxidase activity in these fruits. Furthermore, our fruit proteomics analysis revealed a significant upregulation of SOD-associated proteins in response to SeNPs treatment., and the SOD enzyme in plants is known to neutralize the superoxide radicals and protect cells from oxidative damage (Tian et al., 2021).

In addition, the glutathione S-transferase associated proteins were significantly upregulated in SeNPs-treated diseased fruits. It has been suggested that such an upregulation is a general sign of stress response (Marrs, 1996). These proteins play a critical role in the antioxidant defense system of plants by regulating oxidative stress during bacterial infection (Martinelli et al., 2016). Addition of selenium, a key component of selenocysteine, is known to enhance the efficiency of glutathione S-transferase proteins (Hasanuzzaman and Fujita, 2011). As previously suggested, high citrus susceptibility to HLB may be linked to a failure of antioxidant components to mitigate the deleterious impacts of ROS generated in *C*Las infection (Albrecht and Bowman, 2012). Thus upregulation of glutathione S-transferase associated proteins may explain a part of the positive effect of SeNPs treatment on HLB infected ‘Kinnow’ mandarin plants.

Proteomics analysis also showed that SeNPs upregulated the expression of translation elongation factors and proteins related to structural integrity. This could explain why SeNPs treatment increased total protein content, while untreated diseased plants exhibited lower levels of soluble proteins.

Overall, our results are in line with the findings that silver nanoparticles altered the proteins involved in the stress signaling responses and cellular metabolism (Mustafa et al., 2015). Some other researchers reported that TiO_2_NPs at the 40 mg L^-1^ concentration significantly altered the proteomics expression in wheat plants under biotic stress. The proteins linked to carbohydrate and protein metabolism and photosynthesis were downregulated under biotic stress, while TiO_2_NPs significantly upregulated proteins related to photosynthesis, translation, and plant defense systems, similar to our results (Satti et al., 2022). Our findings are also in line with those of (Mustafa et al., 2015) who described that silver nanoparticles upregulated the proteins related to the translation process in soybean plants. Likewise, Al_2_O_3_ nanoparticles enhanced the abundance of proteins that are involved in protein synthesis (Yasmeen et al., 2016a), while iron nanoparticles enhanced the proteins linked to protein metabolism and photosynthesis (Yasmeen et al., 2016b).

## 5 Conclusions

**Figure 6:**
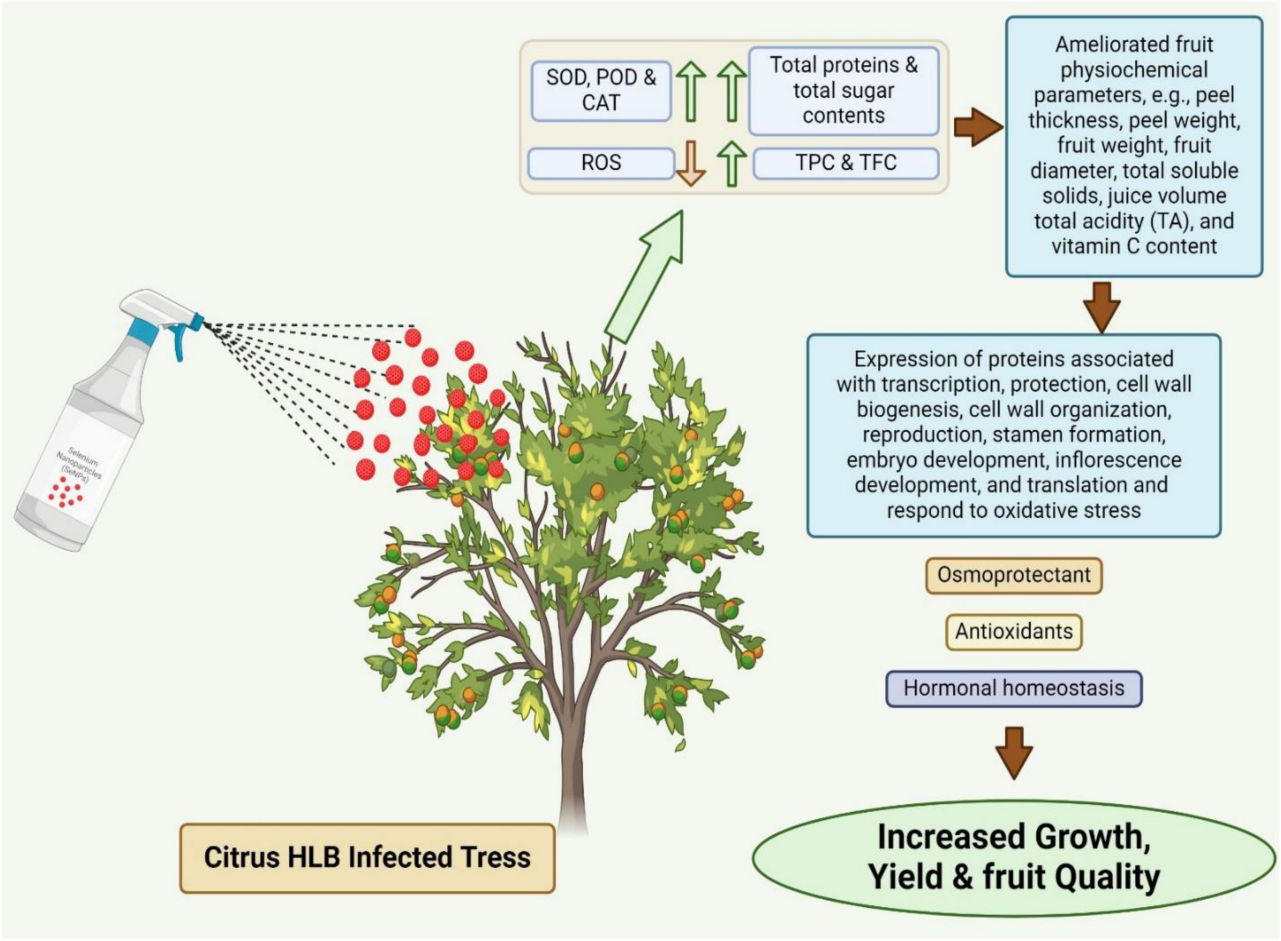
Pictorial overview of possible mechanistic action of SeNPs derived from garlic cloves extract in citrus HLB infected ‘Kinnow’ mandarin trees.

The current study focused on the differential effects of SeNPs on physicochemical, biochemical, and protein expression in ‘Kinnow’ mandarin plants under biotic stress. SeNPs showed dose dependent response up to optimal concentration of 75 mg L^-1^ and the ability to reduce the negative effects of biotic stress. All the tested concentrations have positive impact on the health of HLB infected fruting plants, with 75 mg L^-1^ giving the strongest results. Even lower concentrations of SeNPs significantly improved the fruit quality and physico-biochemical attributes as well as enhanced antioxidant defensive enzymatic activities in HLB-infected ‘Kinnow’ mandarin plants. Pictorial overview of possible mechanistic action of garlic cloves extract mediated SeNPs in citrus HLB infected trees is shown in **Figure 6**.

Proteomic analysis of seeds revealed that NPs significantly affect protein levels, which explains the alternation of fruit physio-chemical and biochemical systems. Finally, it can be concluded that application of SeNPs to plants under biotic stress is an important research area that deserves researchers’ attention due to the important potential applications in horticulture. However, a detailed scientific investigation is still desired to understand the action mechanism of SeNPs. More collaborative efforts and studies are needed to understand the molecular impact of garlic extract mediated SeNPs in curing the HLB disease. Such investigation can significantly facilitate the control of HLB disease in citrus and help the citrus industry worldwide.

## Data Availability

The raw data supporting the conclusions of this article will be made available by the authors, without undue reservation.
