# Supplementary material for: Differential impact of plant-based selenium nanoparticles on physio-biochemical properties, antioxidant defense system and protein regulation in fruits of huanglongbing-infected ‘Kinnow’ mandarin plants
Source: Front Plant Sci. 2024 Nov 7;15:1476497. doi: 10.3389/fpls.2024.1476497 (PMC11578725; doi:10.3389/fpls.2024.1476497)
Supplement: Supplementary file 1 [file DataSheet1.pdf]

Supplementary data table T0 and T1 (seeds)

Proteins	Species name	GOMF name (gene ontology molecular function)	GOBP name (gene ontology biological function)	Normalized Average of (T0)	Normalized Average of (T1)	Ratio	<0.05
A0A067CZL7	*Citrus sinensis*	RNA binding	Translation	674.898671	410.4747338	0.608202	0.032847
V4SY93	*Citrus clementina*	Allene-oxide-cyclase activity	Jasmonic acid metabolism	76.19370993	429.4119554	5.635793	0.025759
V4S392	*Citrus clementina*	Acetyl CoA ligase activity	Lipid metabolism	3602.583861	542.7480342	0.150655	0.006622
V4U519	*Citrus clementina*	Transcription	Inflorescence development	1362.121155	488.6021059	0.358707	0.024214
V4SVQ9	*Citrus clementina*	Unfolded protein binding	Response to stress	316.6874318	40.24471212	0.12708	0.010461
A0A067DQM9	*Citrus sinensis*	Glutathione transferase activity	Response to chemicals	1703.384608	1026.054007	0.602362	0.01354
V4U7H5	*Citrus clementina*	Aminopeptidase activity	Proteolysis	2709.826386	735.7516295	0.271512	0.004021
V4UM01	*Citrus clementina*	Phosphatidylcholine binding	Respond to abiotic stress	1081.372009	505.3433738	0.467317	0.001597
V4TQ66|	*Citrus clementina*	L-malate dehydrogenase activity	Malate metabolic process	157.8588631	337.6395903	2.13887	0.020479
A0A067ERC6	*Citrus sinensis*	Fatty acid synthase activity	Embryo development ending in seed dormancy	387.5954007	129.7744079	0.334819	0.012319
|V4V1N5	*Citrus clementina*	Peptidase activity	Proteolysis	776.0942697	122.3250372	0.157616	0.025581
A0A067FF32	*Citrus sinensis*	Aspartic-type endopeptidase activity	Proteolysis	505.2683114	247.5664137	0.48997	0.024397
V4S535	*Citrus clementina*	Peptidase activity	Stamen formation	590.5157419	375.7515816	0.636311	0.007624
V4TVB1	*Citrus clementina*	NAD binding	Pyruvate metabolic process	190.2177143	26.61051877	0.139895	0.003543
A0A067FH27	*Citrus sinensis*	Serine-type endopeptidase activity	Proteolysis	817.7040915	1673.956752	2.047142	0.02876
A0A067FI88	*Citrus sinensis*	Peroxidase activity	Response to oxidative stress	906.1508578	557.8661977	0.615644	0.021346
V4TS21	*Citrus clementina*	Threonine-type endopeptidase activity	Response to zinc ion	745.3566089	222.393205	0.298372	0.034178
X2J355	*Citrus sinensis*	Cysteine-type peptidase activity	Proteolysis	454.9558064	1944.133634	4.273236	0.01476
A0A067GH18	*Citrus sinensis*	uncharacterized	Uncharacterized	1703.167693	666.1498671	0.391124	0.018759
A0A067GNU4	*Citrus sinensis*	GTPase activity	Protein transport	390.4167473	75.7169092	0.193939	0.012215
V4TE12	*Citrus clementina*	uncharacterized	Uncharacterized	173.4353781	21.39372255	0.123353	0.017187
A0A067H9U2	*Citrus sinensis*	Glucose -6-phosphate isomerase activity	Gluconeogenesis	291.5991414	61.04391212	0.209342	0.047286
A0A067GYQ8	*Citrus sinensis*	Protein disulfide isomerase activity	Respond to endoplasmic reticulum stress	3691.447463	1974.004265	0.534751	0.042239
A0A067H0C3	*Citrus sinensis*	phosphoprotein phosphatase	Inflorescence development	162.7231602	40.94912623	0.251649	0.014853
A0A1S8ACJ4	*Citrus lemon*	ATP dependent protein folding chaperon	Protein refolding	1100.287118	191.5143884	0.174059	0.012024

V4WGV2	*Citrus clementina*	ATP hydrolysis activity	Protein folding	1153.021142	467.9150962	0.405817	0.044779
Q39629	*Citrus sinensis*	Acetyl CoA ligase activity	Reproduction	4131.578122	484.1529696	0.117184	0.028555
V4RJK9	*Citrus clementina*	Adenosylhomocysteinase activity	Carbon metabolism	1264.228029	295.143958	0.233458	0.019658
V4RMN0	*Citrus clementina*	Acireductone synthase activity	Proline metabolism	6970.880432	17574.8086	2.521175	0.026742
V4RNH3	*Citrus clementina*	ATP-dependent protein folding chaperone	Response to stress	662.3027895	166.1530355	0.250872	0.032308
V4S7V0	*Citrus clementina*	uncharacterized	Uncharacterized	317.4810637	67.07950336	0.211287	0.02327
V4SGH6	*Citrus clementina*	Calcium ion binding	Protein folding	438.8366028	94.48341543	0.215304	0.019956
V4SJT5	*Citrus clementina*	ATP dependent protein folding chaperon	Respond to stress	8333.119941	4565.407361	0.547863	0.036855
V4SKG4	*Citrus clementina*	Glucose -6-phosphate isomerase activity	Gluconeogenesis	1156.94605	534.7665238	0.462223	0.032278
V4SMC0	*Citrus clementina*	Catalytic activity	Cell wall organization	1612.665132	533.7130373	0.330951	0.006374
V4SR38	*Citrus clementina*	Actin filament binding	Cell wall biogenesis	32.10010495	5.945904983	0.18523	0.232827
V4STL6	*Citrus clementina*	Protein self-Association	Response to heat	1209.406474	356.3578128	0.294655	0.001179
V4SXD5	*Citrus clementina*	Hydrolase activity	Cell organization	806.4510684	362.8179169	0.449895	0.005862
V4SZI9	*Citrus clementina*	Metalloendopeptidase activity	Mitochondrial transport	273.2684224	65.32676063	0.239057	0.012516
V4T992	*Citrus clementina*	Transaminase activity	Peptide catabolic process	53.80485791	276.8834309	5.146068	0.009143
V4TAG2	*Citrus clementina*	Glutamate 5-Kinase activity	Proline biosynthesis	96.68753501	288.8227592	2.987177	0.004148
V4TKF6	*Citrus clementina*	Super oxide dismutase activity	Protection	783.2077763	73.66237122	0.094052	0.001126
V4THB5	*Citrus clementina*	Oxidoreductase activity	Cell redox homeostasis	430.3850604	50.83830083	0.118123	0.001336
V4TNQ8	*Citrus clementina*	Carbon-carbon lyase activity	Gluconeogenesis	362.942687	88.95401848	0.245091	0.010342
V4TRS7	*Citrus clementina*	NADP binding	Glucose metabolism	286.6413885	148.5048463	0.518086	0.030488
V4TX11	*Citrus clementina*	Protein self-association	Transcription	285.0084396	149.12976	0.523247	0.038801
V4TZU5	*Citrus clementina*	Oxidoreductase activity	Glycolytic process	591.7676406	229.2730033	0.387438	0.005414
V4UGU0	*Citrus clementina*	Chitinase activity	Carbohydrate metabolism	540.6284962	260.0123611	0.480945	0.031902
V4UHG4	*Citrus clementina*	uncharacterized	uncharacterized	1871.141323	752.3450769	0.402078	0.020648
V4UI07	*Citrus clementina*	Structural constituents of ribosomes	Translation	352.969473	65.63580892	0.185953	0.007055
V4UJ63	*Citrus clementina*	Uncharacterized	Uncharacterized	240.0993901	38.21075243	0.159146	0.027232
V4UJF9	*Citrus clementina*	Oxidoreductase activity	Primary metabolic process	785.295022	4586.545313	5.840538	0.04422
V4W9L3	*Citrus clementina*	Hydrolase activity	Small molecule catabolic process	440.854721	1467.153511	3.327975	0.043453
V4S392	Citrus clementina	Acetyl CoA ligase activity	Reproduction	3602.583861	946.9844729	0.262863	0.016324
A0A067DC44	*Citrus sinensis*	hydrolase activity	cell wall organization	187.127645	112.3265768	0.600267	0.025142

T0 and T5 (Seeds)

Proteins	Species name	GOMF name (gene ontology molecular function)	GOBP name (gene ontology biological function)	Normalized Average of T0	Normalized Average of T5	Ratio	P<0.05
A0A067D3A9	*Citrus sinensis*	Uncharacterized protein	Uncharacterized protein	566.6918133	244.7446391	0.431883139	0.010164215
V4S392	*Citrus clementina*	Fatty acid synthase activity	Lipid biosynthetic process	917.2934438	3602.583861	3.927406094	0.015278158
A0A067EBT2	*Citrus sinensis*	Translation elongation factor activity	Protein biosynthesis	1049.3061	1789.248414	1.705172985	0.03683252
A0A067ECI6	*Citrus sinensis*	Uncharacterized protein	Uncharacterized protein	70.66606868	12.11843817	0.171488784	0.020708577
V4TQ66	*Citrus clementina*	L-malate dehydrogenase activity	Malate metabolic process	337.6395903	177.9702549	0.527101264	0.049276154
V4V1N5	*Citrus clementina*	Aspartic-type endopeptidase activity	Proteolysis	776.0942697	243.95394	0.314335448	0.020468769
A0A067FI88	*Citrus sinensis*	Peroxidase activity	Response to oxidative stress	520.9812026	906.1508578	1.73931584	0.016405294
V4T6T5	*Citrus clementina*	RNA binding	Translation	130.4054285	281.5694223	2.159184825	0.034020461
X2J355	*Citrus sinensis*	Cysteine type-endopeptidase activity	Proteolysis	1944.133634	920.5473452	0.473500036	0.045394726
V4VJX2	*Citrus clementina*	RNA binding	rRNA processing	141.0853061	206.5406148	1.46394136	0.044220659
A0A067G7D1	*Citrus sinensis*	Pectin esterase activity	Cell wall modification	120.1962861	306.0742074	2.546453116	0.017602217
Q948T8	*Citrus jambhiri*	DNA binding	Structural constituents of chromatin	437.5522582	242.1050559	0.553316893	0.0019717
A0A067GGT9	*Citrus sinensis*	Uncharacterized protein	Uncharacterized protein	1586.13639	906.9719654	0.571812091	0.047258074
A0A067GNU4	*Citrus sinensis*	GTPase activity	Transport	148.4048687	390.4167473	2.630754307	0.001409839
tr|A0A1S8ACJ4	*Citrus limon*	ATP binding	Protein refolding	1100.287118	489.0628759	0.444486596	0.010276047
Q39629	*Citrus sinensis*	acyl-CoA ligase activity	Lipid metabolism	994.0126189	1131.578122	1.138394122	0.036386614
V4RJK9	*Citrus clementina*	Adenosylhomocysteinase activity	Carbon metabolism	1264.228029	628.0658357	0.496797905	0.032276137
V4SQX0	*Citrus clementina*	Uncharacterized protein	Uncharacterized protein	600.2018385	103.1634897	0.171881329	0.029614637
V4T939	*Citrus clementina*	Serine-type carboxypeptidase activity	proteolysis	840.9145816	595.436932	0.708082539	0.022837573
V4TAG2	*Citrus clementina*	Uncharacterized protein	Uncharacterized protein	288.8227592	88.90763484	0.307827663	0.045871708
V4UGU0|	*Citrus clementina*	Chitinase activity	chitin metabolism	540.6284962	305.5521285	0.565179473	0.041755099
V4VPK9	*Citrus clementina*	Uncharacterized protein	Uncharacterized protein	119.848813	68.22415967	0.56925186	0.025141813
V4WEE3	*Citrus clementina*	RNA binding	translation	89.1253435	177.128318	1.987406848	0.025141813
tr|V4SRE5	*citrus clementina*	dihydroneopterin aldolase activity	Folic acid metabolism	761.123675	970.325435	1.274859089	0.025141813
V4W5U2	*citrus clementina*	Phosphoglucomutase activity	glucose metabolic processes	680.135676	876.986787	1.28942918	0.025141813
V4WDK0	*citrus clementina*	Metalloendopeptidase activity	Proteolysis	970.1343265	1143.185645	1.178378719	0.025141813

V4TJQ9	Citrus clementina	Hsp90 protein binding	Protein folding	1188.965896	237.2742099	0.199563512	0.056760823
A0A8A4JGR3	Citrus clementina	ATP binding	Transport	153.1059813	32.25354241	0.210661544	0.045382347
V4SY93	Citrus clementina	Allene-oxide cyclase activity	Jasmonic acid biosynthesis	429.4119554	687.5754602	1.601202415	0.057441124
V4RVN7	Citrus clementina	Metal ion binding	Response to water deprivation	1286.001184	8239.829923	6.407326858	0.03120685
A0A067DQM9	Citrus sinensis	Glutathione transferase activity	Translation and detoxification	1703.384608	3467.206316	2.035480595	0.010029585
A0A067ERS2	Citrus sinensis	Calcium Ion binding	Response to stress	311.3414301	527.9223192	1.695637869	0.014823472
V4V1N5	Citrus clementina	Aspartic -type endopeptidase activity	Proteolysis	776.0942697	244.0889406	0.314509397	0.035885506
V4S886	Citrus sinensis	structural constituents of Ribosomes	Translation	167.8603174	275.5708134	1.641667416	0.021506383
A0A067FF32	Citrus sinensis	Aspartic -type endopeptidase activity	lipid metabolic process	505.2683114	817.119407	1.617198998	0.032456077
V4TVB1	Citrus sinensis	Metal ion binding	Malate metabolic process	190.2177143	61.64387839	0.324070125	0.010528528
A0A067FI92	Citrus sinensis	Peroxidase activity	Response to pathogen attack	1759.260274	3641.258607	2.069766856	0.032066668
V4RU72	Citrus clementina	Pyruvate dehydrogenase activity	Glycolytic activity	245.5378081	1386.038503	5.644908674	0.034441972
A0A067H799	Citrus sinensis	Peroxidase activity	hydrogen peroxide removal	192.8866032	422.0764681	2.188210384	0.009515247
V4T0S4	Citrus clementina	Uncharacterized protein	Uncharacterized protein	185.1942727	1338.364839	7.226815489	0.022257865
V4TBA1	Citrus clementina	Pyruvate dehydrogenase activity	Unfold protein binding	11613.30696	4285.043322	0.368977014	0.010009576
V4TJQ9	Citrus clementina	Hsp90 protein binding	Protein folding	1176.953896	1237.17421	1.051166247	0.056760823
V4TW14	Citrus clementina	Protein disulfide isomerase activity	Pollen tube development	227.9584737	517.4577907	2.269965149	0.012973509
V4UBR6	Citrus clementina	Catalytic activity	Cell wall organization	798.4360781	1440.265576	1.803858337	0.03358495
V4UDD5	Citrus clementina	Phosphoric ester hydrolase activity	Response to stimulus	215.0266384	773.7234101	3.598267712	0.008926967
V4VLH3	Citrus clementina	Endopeptidase activity	Protein metabolic process	264.882938	32.05331295	0.121009353	0.02216114
V4S392	Citrus clementina	Acetyl CoA ligase activity	Reproduction	3104.512456	5154.984473	1.660481169	0.016323501
V4S535	*Citrus clementina*	Peptidase activity	Stamen formation	375.7515816	590.5157419	1.571558899	0.007623535
A0A067H0C3	*Citrus sinensis*	phosphoprotein phosphatase	Inflorescence development	45.94912623	172.7231602	3.759008589	0.014852646
Q39629	*Citrus sinensis*	Acetyl CoA ligase activity	Reproduction	384.1529696	1131.578122	2.945644604	0.028555157
V4TKF6	*Citrus clementina*	Superoxide dismutase activity	Protection	73.66237122	283.2777631	3.845623734	0.001125999
